# Hyperinsulinemia correlates with low levels of plasma B-type natriuretic peptide in Japanese men irrespective of fat distribution

**DOI:** 10.1186/1475-2840-11-22

**Published:** 2012-03-07

**Authors:** Hideaki Nakatsuji, Ken Kishida, Tohru Funahashi, Tohru Nakagawa, Iichiro Shimomura

**Affiliations:** 1 Department of Metabolic Medicine, Graduate School of Medicine, Osaka University, Suita, Osaka 565-0871, Japan; 2 Department of Metabolism and Atherosclerosis, Graduate School of Medicine, Osaka University, Suita, Osaka 565-0871, Japan; 3 Hitachi, Ltd. Hitachi Health Care Center, Hitachi, Ibaraki 317-0076, Japan; 4 Department of Metabolism and Atherosclerosis, Graduate School of Medicine, Osaka University, 2-2 B-5, Yamada-oka, Suita, Osaka 565-0871, Japan

**Keywords:** B-type natriuretic peptide, Hyperinsulinemia, Visceral fat

## Abstract

**Background:**

B-type natriuretic peptide (BNP), a member of the natriuretic peptide family, is a cardiac-derived secretory hormone with natriuretic, diuretic, and vasorelaxant activities. Intraabdominal fat accumulation is associated with atherosclerotic cardiovascular diseases and cardiac dysfunction. Circulating BNP levels are relatively low (within the normal limits) in obesity and the metabolic syndrome. However, the relationship between plasma BNP levels and visceral fat accumulation in general population has not been reported. The present study analyzed the relationships between plasma BNP levels and various clinical variables, including insulin, visceral and subcutaneous fat area (VFA and SFA, respectively), in normal Japanese men.

**Methods:**

The study (Victor-J study) subjects were consecutive 500 Japanese male workers, who underwent a health checkup and were measured VFA and SFA by computed tomography.

**Results:**

Age-adjusted simple linear regression analysis showed that log-BNP correlated positively with HDL-cholesterol, and negatively with VFA, log-immunoreactive insulin (IRI), log-triglyceride, and LDL-cholesterol, but not body mass index or SFA. Stepwise multiple regression analysis identified log-IRI and HDL-cholesterol as significant determinants of log-BNP. Subjects with IRI ≥5.5 μIU/mL had lower plasma BNP levels than those with IRI < 5.5 μIU/mL, irrespective of obesity (body mass index, cutoff value 25 kg/m^2^), visceral fat accumulation (VFA, cutoff value 100 cm^2^) and subcutaneous fat accumulation (SFA, cutoff value 128 cm^2^).

**Conclusions:**

Our study showed that hyperinsulinemia correlated with low levels of plasma BNP in general men, irrespective of fat distribution.

**Trial registration:**

UMIN 000004318.

## Background

B-type natriuretic peptide (BNP), a member of the natriuretic peptide (NP) family, is a cardiac-derived secretory hormone with natriuretic, diuretic, and vasorelaxant activities [1,2]. Plasma BNP level correlates with the severity of heart failure, and is clinically used as a marker of cardiac dysfunction [3]. Evidence suggests that high BNP levels regulate endogenous antagonism of vasoconstriction, the salt- and water-retaining system that acts to prevent a rise in blood pressure, and plasma volume expansion through direct natriuresis, diuresis, and vasodilation.

Visceral fat accumulation correlated closely with systolic blood pressure [4], and is also related to the development of cardiac dysfunction [5]. However, circulating NPs levels are relatively low (within the normal limits) in obesity [6-11]. It has been suggested that obese subjects have a "natriuretic handicap", with a reduced NPs response to cardiac wall stress. Low NPs levels may contribute to the pathophysiology of cardiac dysfunction in visceral adiposity. The present study analyzed the relationships between plasma BNP levels and various clinical variables, including insulin, visceral and subcutaneous fat area (VFA and SFA, respectively), in normal Japanese men.

## Methods

### Participants

The study subjects were consecutive 500 Japanese male employees, who underwent a health checkup in year 2010 at Hitachi Ltd, Ibaraki Prefecture, including computed tomography (CT). The present study was approved by the human ethics committees of the National Center for Global Health and Medicine, Osaka University and Hitachi Health Care Center. Written informed consent was obtained from all subjects. This trial (Victor-J study) is registered with the University Hospital Medical Information Network (#UMIN 000004318)

https://upload.umin.ac.jp/cgi-openbin/ctr/ctr.cgi?function=brows&action=brows&type=summary&recptno=R000005173&lang uage=E.

### Anthropometry and laboratory measurements

Height and weight were measured in standing position. Body mass index (BMI) was calculated and expressed in kg/m^2^. Blood pressure was measured in a sitting position with a standard mercury sphygmomanometer on the right or left arm after the subjects had rested for at least 5 minutes. VFA and SFA were computed and measured automatically using commercial software on a CT scan taken at the umbilical level in supine position [120 kV, 400 mAsec, section thickness of 5-10 mm, field of view of 400 mm, window width of 800- 1,000 Hounsfield units]. Venous blood samples were collected in the morning after overnight fast for measurements of creatinine, hemoglobin A1c (HbA1c), HDL-cholesterol, LDL-cholesterol, triglyceride, glucose, and immunoreactive insulin (IRI). The value for HbA1c (%) is estimated as National Glycohemoglobin Standardization Program (NGSP) equivalent value (%) calculated by the formula HbA1c (%) = HbA1c (Japan Diabetes Society [JDS],%) + 0.4%. Plasma BNP concentrations were measured with specific immunoradiometric assay for human BNP (BNP kit, Shionogi, Osaka, Japan, normal range; < 18.4 pg/mL, intracoefficients of variation (CV); 2.7-9.6%, inter-CV 5.6-11.8%, range [2.0-2,000 pg/mL]).

### Statistical analysis

Data are presented as mean ± SD (Tables) or mean ± SEM (Figure). Stepwise multiple regression analysis was first conducted to identify those parameters that significantly contributed to log-BNP, and parameters with *F *value > 4.0 were subsequently entered into the regression analysis as independent variables. The subjects were divided into four groups according to; (analysis 1) BMI (cutoff value 25 kg/m^2^; obesity) and IRI (cutoff value 5.5 μIU/mL; median value), (analysis 2) VFA (cutoff value 100 cm^2^; visceral fat accumulation) and IRI (cutoff value 5.5 μIU/mL; median value), (analysis 3) SFA (cutoff value 128 cm^2^; median value) and IRI (cutoff value 5.5 μIU/mL; median value). Differences among groups were compared by one- or two-way analysis of variance (ANOVA) with Fisher's protected least significant difference test for multiple-group analysis or unpaired Student's *t*-test for experiments involving only two groups. In all cases, *p *values < 0.05 were considered statistically significant. All analyses were performed with the JMP Statistical Discovery Software 8.0 (SAS Institute, Cary, NC).

## Results

### Characteristics of subjects enrolled in the present study

The baseline characteristics of the subjects who underwent a health checkup are listed in Table 1. Only 12.4% (n = 62) were hyper-BNPemic (above the normal range; upper limit of normal range; ≥18.4 pg/mL), and 70.2% of the subjects (n = 351) had visceral fat accumulation (VFA ≥100 cm^2^).

**Table 1:** Baseline characteristics of male subjects participating in the present study (n = 500)

	mean ± SD [range or n (%)]
Age, years	55 ± 9 (30-74)
Body mass index (BMI), kg/m^2^	24.2 ± 3.0 (14.8-36.5)
Visceral fat area (VFA), cm^2^	127 ± 54 (3-294)
Subcutaneous fat area (SFA), cm^2^	136 ± 56 (5-361)
Blood glucose, mg/dL	110 ± 18 (78-284)
Fasting immunoreactive insulin (IRI), μIU/mL	6.7 ± 4.5 (0.6-42.3)
HbA1c (NGSP),%	5.8 ± 0.6 (4.6-10.2)
Systolic blood pressure (SBP), mmHg	123 ± 12 (91-174)
Diastolic blood pressure (DBP), mmHg	78 ± 8 (47-100)
Triglyceride, mg/dL	140 ± 95 (35-871)
High-density lipoprotein cholesterol (HDL-cholesterol), mg/dL	56 ± 13 (32-97)
Low-density lipoprotein cholesterol (LDL-cholesterol), mg/dL	122 ± 28 (39-199)
Estimated glomerular filtration rate (eGFR), mL/min	73.7 ± 12.8 (41.9-128.5)
Plasma B-type natriuretic peptide (BNP), pg/mL	10.8 ± 13.0 (2.0-159)
Smoking (none/ex-/current-smoker)	131/240/129
Diabetes mellitus (under medication)	24 (17)
Hypertension (under medication)	116 (115)
Dyslipidemia (under medication)	63 (59)
Past history of CAD/CVD	5/3

CAD, coronary artery disease, CVD, Cerebrovascular disease

Glomerular filtration rate was estimated by eGFR = 194 × serum creatinine^-1.094 ^× age^-0.287^

### Correlation analysis between plasma BNP levels and measured parameters

**Figure 1:**
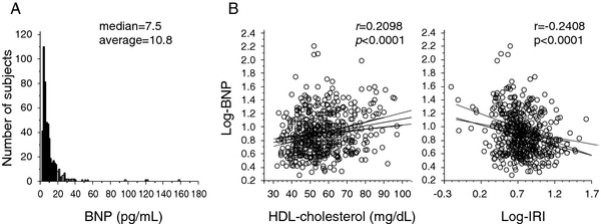
**A histogram of plasma BNP levels and correlation between log-IRI, HDL-cholesterol and log-BNP**. Pearson's correlation analysis was used to examine the relationships between log-IRI, HDL-cholesterol and log-BNP. Regression curve with 95% confidence interval of predicted mean together with reference lines indicating baseline (0% change)

Table 2 lists the correlation coefficients for the relationship between BNP and various clinical parameters. Data of BNP showed skewed distribution (Figure 1A), and therefore were log-transformed before analysis. Age-adjusted simple linear regression analysis showed that log-BNP correlated positively with HDL-cholesterol (Figure 1B), and negatively with VFA, log-IRI (Figure 1B), log-triglyceride, and LDL-cholesterol, but not BMI or SFA. Stepwise multiple regression analysis identified HDL-cholesterol and log-IRI as significant and independent determinants of log-BNP.

**Table 2:** Results of simple and stepwise multiple regression analyses for log-BNP

	Simple (non-adjusted)		Simple (age-adjusted)		Multiple
	***r***	***p***	***r***	***p***	***F *value**
Age	0.3688	< 0.0001	-	-	
BMI	-0.1225	0.0069	-0.0080	0.0559	
VFA	-0.0707	0.1037	-0.0010	0.0048	0.007
SFA	-0.1342	0.0025	-0.0004	0.0816	
Blood glucose	0.0837	0.0579	0.0003	0.7088	
Log-IRI	-0.2408	< 0.0001	-0.2430	< 0.0001	11.771
HbA1c	0.0894	0.0527	0.0050	0.8280	
Systolic blood pressure	0.1304	0.0031	0.0010	0.2595	
Diastolic blood pressure	-0.0134	0.7650	0.0020	0.2815	
Log-triglyceride	-0.1517	0.0007	-0.1480	0.0079	0.385
HDL-cholesterol	0.2098	< 0.0001	0.0040	< 0.0001	6.437
LDL-cholesterol	-0.1414	0.0014	-0.0010	0.0088	2.377
eGFR	-0.1304	0.0035	-0.0004	0.6988	
Smoking (ex-+current-)	-0.0088	0.8442	-0.0090	0.7565	

Data of BNP, IRI and triglyceride levels showed skewed distribution, and therefore were log-transformed before analysis. Multiple; [adopted factors: age, VFA, log-IRI, log-triglyceride, HDL-cholesterol, LDL-cholesterol]

### Comparisons of clinical features including plasma BNP levels according to obesity and fat distribution

**Figure 2:**
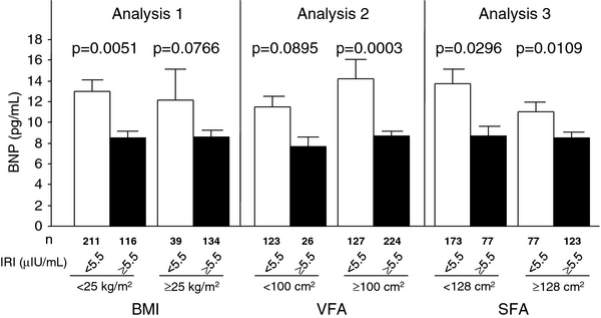
**Comparison of plasma BNP levels between subjects without and with hyperinsulinemia (IRI cutoff value 5.5 μIU/mL; median value), and obesity (BMI cutoff value 25 kg/m^2^, Analysis 1), visceral fat accumulation (VFA cutoff value 100 cm^2^, Analysis 2) or subcutaneous fat accumulation (SFA cutoff value 128 cm^2^; median value, Analysis 3)**. Data are mean ± SEM.

Subjects with IRI ≥5.5 μIU/mL had lower plasma BNP levels than those with IRI < 5.5 μIU/mL, irrespective of obesity (analysis 1, Table 3), visceral fat accumulation (analysis 2, Table 4), and subcutaneous fat accumulation (analysis 3, Table 5) (Figure 2). These results suggest that hyperinsulinemia correlates with low levels of plasma BNP, irrespective of fat distribution.

**Table 3:** Comparison of clinical features between subjects without and with obesity (BMI cutoff value 25 kg/m^2^) and hyperinsulinemia (IRI cutoff value 5.5 μIU/mL; median value) (Analysis 1) (n = 500)

	BMI < 25 kg/m^2^		BMI ≥ 25 kg/m^2^	
	**IRI < 5.5 μIU/mL**	**IRI ≥ 5.5 μIU/mL**	**IRI < 5.5 μIU/mL**	**IRI ≥ 5.5 μIU/mL**
Age, years	55 ± 9	54 ± 10	55 ± 8	53 ± 9
BMI, kg/m^2^	22.1 ± 1.8	23.2 ± 1.2*	26.2 ± 1.5*	27.6 ± 2.4^†§ ^
VFA, cm^2^	94 ± 44	131 ± 40*	144 ± 36*	171 ± 45^†§^
SFA, cm^2^	103 ± 36	127 ± 33*	154 ± 42*	191 ± 58^†§^
Blood glucose, mg/dL	106 ± 15	114 ± 20*	114 ± 33*	111 ± 13
Fasting IRI, μIU/mL	3.8 ± 1.1	7.8 ± 2.6*	4.5 ± 0.8*	11.0 ± 5.8^†§^
HbA1c (NGSP),%	5.7 ± 0.5	5.8 ± 0.7*	6.0 ± 0.9*	5.8 ± 0.5
SBP, mmHg	120 ± 12	124 ± 11*	126 ± 11*	125 ± 10
DBP, mmHg	76 ± 8	79 ± 7*	78 ± 14	80 ± 7
Triglyceride, mg/dL	117 ± 88	148 ± 99*	171 ± 112*	161 ± 89
HDL-cholesterol, mg/dL	61 ± 13	55 ± 12*	54 ± 8*	51 ± 11^†^
LDL-cholesterol, mg/dL	117 ± 28	121 ± 27	124 ± 30	131 ± 27^†^
eGFR, mL/min	74 ± 13	76 ± 13	74 ± 11	71 ± 13^†^

Data are mean ± SD

*p < 0.01, compared to with BMI < 25 kg/m^2 ^and IRI < 5.5 μIU/mL

^†^p < 0.01, compared to with BMI < 25 kg/m^2 ^and IRI ≥ 5.5 μIU/mL

^§^p < 0.01 compared to with BMI ≥ 25 kg/m^2 ^and IRI < 5.5 μIU/mL

**Table 4:** Comparison of clinical features between subjects without and with visceral fat accumulation (VFA cutoff value 100 cm^2^) and hyperinsulinemia (IRI cutoff value 5.5 μIU/mL; median value) (Analysis 2) (n = 500)

	VFA < 100 cm^2^		VFA ≥ 100 cm^2^	
	**IRI < 5.5 μIU/mL**	**IRI ≥ 5.5 μIU/mL**	**IRI < 5.5 μIU/mL**	**IRI ≥ 5.5 μIU/mL**
Age, years	53 ± 11	47 ± 12*	56 ± 8*	54 ± 9^†§ ^
BMI, kg/m^2^	21.6 ± 2.0	23.1 ± 2.5*	23.9 ± 2.0*	25.9 ± 2.8^†§^
VFA, cm^2^	63 ± 26	72 ± 24	139 ± 28*	162 ± 40^†§^
SFA, cm^2^	92 ± 38	114 ± 58*	129 ± 37*	167 ± 55^†§^
Blood glucose, mg/dL	104 ± 14	110 ± 14	110 ± 23*	113 ± 17
Fasting IRI, μIU/mL	3.6 ± 1.1	7.1 ± 1.4*	4.2 ± 0.9*	9.8 ± 5.0^†§^
HbA1c (NGSP),%	5.6 ± 0.5	5.6 ± 0.5	5.8 ± 0.6*	5.9 ± 0.6
SBP, mmHg	118 ± 12	121 ± 13	123 ± 11*	125 ± 10
DBP, mmHg	75 ± 9	77 ± 7	78 ± 10*	80 ± 7^§^
Triglyceride, mg/dL	101 ± 63	108 ± 49	149 ± 111*	160 ± 96^†^
HDL-cholesterol, mg/dL	62 ± 13	57 ± 10	58 ± 13*	52 ± 12^†§^
LDL-cholesterol, mg/dL	115 ± 29	124 ± 25	121 ± 28	127 ± 28
eGFR, mL/min	76 ± 12	79 ± 13	73 ± 12	73 ± 13^†^

Data are mean ± SD

*p < 0.01, compared to with VFA < 100 cm^2 ^and IRI < 5.5 μIU/mL

^†^p < 0.01, compared to with VFA < 100 cm^2 ^and IRI ≥ 5.5 μIU/mL

^§^p < 0.01 compared to with VFA ≥ 100 cm^2 ^and IRI < 5.5 μIU/mL

**Table 5:** Comparison of clinical features between subjects without and with subcutaneous fat accumulation (SFA cutoff value 128 cm^2^; median value) and hyperinsulinemia (IRI cutoff value 5.5 μIU/mL; median value) (Analysis 3) (n = 500)

	SFA < 128 cm^2^		SFA ≥128 cm^2^	
	**IRI < 5.5 μIU/mL**	**IRI ≥5.5 μIU/mL**	**IRI < 5.5 μIU/mL**	**IRI ≥5.5 μIU/mL**
Age, years	55 ± 9	55 ± 10	55 ± 9	52 ± 9^†^
BMI, kg/m^2^	21.9 ± 1.9	23.4 ± 1.9*	24.7 ± 1.9*	26.6 ± 2.8^†§^
VFA, cm^2^	88 ± 43	128 ± 48*	131 ± 41*	164 ± 43^†§^
SFA, cm^2^	91 ± 27	104 ± 21*	157 ± 31*	187 ± 50^†§^
Blood glucose, mg/dL	106 ± 20	115 ± 19*	108 ± 16	111 ± 16
Fasting IRI, μIU/mL	3.7 ± 1.1	7.4 ± 1.9*	4.4 ± 0.9*	10.4 ± 5.4^†§^
HbA1c (NGSP),%	5.7 ± 0.5	5.9 ± 0.7*	5.8 ± 0.6	5.8 ± 0.5
SBP, mmHg	120 ± 13	126 ± 13*	123 ± 11*	124 ± 10
DBP, mmHg	76 ± 8	79 ± 7*	78 ± 11	80 ± 7
Triglyceride, mg/dL	117 ± 81	157 ± 114*	145 ± 117*	153 ± 83
HDL-cholesterol, mg/dL	62 ± 13	55 ± 11*	56 ± 12*	51 ± 12^†§^
LDL-cholesterol, mg/dL	119 ± 31	124 ± 30	118 ± 24	128 ± 26^§^
eGFR, mL/min	75 ± 13	74 ± 15	73 ± 11	73 ± 12

Data are mean ± SD

*p < 0.01, compared to with SFA < 128 cm^2 ^and IRI < 5.5 μIU/mL

^†^p < 0.01, compared to with SFA < 128 cm^2 ^and IRI ≥5.5 μIU/mL

^§^p < 0.01 compared to with SFA ≥128 cm^2 ^and IRI < 5.5 μIU/mL

## Discussion

Our cross-sectional study of Japanese male subjects demonstrated for the first time that hyperinsulinemia correlated with low levels of plasma BNP, irrespective of fat distribution. The present study also found that HDL-cholesterol was a significant determinant of log-BNP. Although Wang et al. demonstrated that reduced HDL-cholesterol was associated with lower plasma BNP levels by multivariable analysis [12], the mechanism remains unclear.

As the mechanisms, we should consider the effect of hyperinsulinemia on BNP production and catabolism. There is controversy on whether or not hyperinsulinemia directly suppresses BNP production [13,14]. NPs, through the activation of the biologically active membrane guanylate-cyclase-linked NPR-1 has a potent lipolytic effect in human adipocytes via a cGMP-dependent mechanism [15] and activation of hormone-sensitive lipases [16,17]. Metabolism of NPs is regulated by two degradative pathways; uptake by the clearance receptor, natriuretic peptide receptor-3 (NPR-3) and hydrolysis by NEP [18]. Our and the other groups previously demonstrated that, 1) NPR-1, NPR-2 and NPR-3 mRNAs are expressed in the adipose tissues, however mRNA level of NEP is relatively low [19], and 2) lower NPR-1 mRNA levels and higher NPR-3 mRNA levels in the adipose tissues of *ob/ob *mice [19] or in the adipose tissues and muscle of diet-induced mice [20], models of hyperinsulinemia, compared to lean mice. Plasma NEP levels were higher in diet-induced mice and tissue NEP was increased in mesenteric fat in diet-induced mice, compared with normal-diet mice [21]. The effect of insulin on NEP has not been reported. Taken together, dysregulation of two degradative pathways under hyperinsulinemia may be, at least partly, responsible for low circulating BNP levels.

Amino-terminal pro-BNP (NT-proBNP), which is co-secreted in equimolar amounts with BNP from the cardiac ventricle, is also well-established as a diagnostic marker in heart failure [22,23]. Surgical weight loss with reduced insulin levels was associated with increases in NT-proBNP [24]. Women with gestational diabetes mellitus under insulin therapy had lower circulating NT-proBNP levels than those under medical nutrition therapy or healthy pregnancies [25]. These results suggest that circulating levels of NT-proBNP as well as BNP may be regulated by insulin. However, clearance of plasma NT-proBNP occurred across kidney, liver, musculoskeletal, and head and neck tissue [26], differ from plasma BNP cleared by NPRs and NEP. Further experimental and clinical studies including both BNP and NT-proBNP are required. Improvement of hyperinsulinemia may reduce plasma volume and thus lower blood pressure through the increase in plasma NPs levels. This may reduce susceptibility for cardiac dysfunction in subjects with hyperinsulinemia.

## Conclusion

In conclusion, hyperinsulinemia correlated with low levels of plasma BNP in general men, irrespective of fat distribution.

### Study limitations

Several limitations of this study must be considered. First, this is a cross-sectional study, making it difficult to establish a cause-effect relationship. Further prospective studies should be conducted in the future to analyze this relationship. Second, the results may not be applicable to females or non-Japanese populations. Finally, in the present study, drug information was based on information provided by the subjects or their relatives. The current study may include the effects of use of various medications for diabetes, hypertension and dyslipidemia. Further studies that include untreated patients need to be conducted.

## Abbreviations

BNP: B-type natriuretic peptide; CT: Computed tomography; IRI: Immunoreactive insulin; NEP: Neutral endopeptidase; NP: Natriuretic peptide; NPR: Natriuretic peptide receptor; SFA: Subcutaneous fat area; VFA: Visceral fat area.

## Competing interests

K.K. and T.F. are members of the "Department of Metabolism and Atherosclerosis", a sponsored course endowed by Kowa Co. Ltd. and a company researcher is dispatched to the course. All other authors declare no competing interests.

## Authors' contributions

HN and KK researched and analyzed data. KK also participated in the concept and design of the study, interpretation of data and reviewed/edited the manuscript. TN recruited the patients and collected the data. TF and IS contributed to discussion and wrote the manuscript. All authors read and approved the final version of the manuscript.
